# Preparation and pharmacokinetic evaluation of *Staphylococcus* phage COP-80B for treatment of periprosthetic joint infections in a mouse model

**DOI:** 10.1016/j.virusres.2025.199592

**Published:** 2025-05-31

**Authors:** Vida Štilec, Monika Marušić, Nika Janež, Urban Bezeljak, Lucija Rebula, Maja Leskovec, Rihard Trebše, Simon Horvat, Matjaž Peterka

**Affiliations:** a COBIK, Mirce 21, 5270 Ajdovščina, Slovenia; b Department of Animal Science, Biotechnical Faculty, University of Ljubljana, Groblje 3, 1230 Domžale, Slovenia; c Sferogen d.o.o, Vipavska cesta 2c, 5270 Ajdovščina, Slovenia; d Sartorius BIA Separations d.o.o., Mirce 21, 5270 Ajdovščina, Slovenia; e Valdoltra Orthopaedic Hospital, Jadranska cesta 31, 6280 Ankaran, Slovenia; f Faculty of Medicine, University of Ljubljana, Vrazov trg 2, 1000 Ljubljana, Slovenia

**Keywords:** Phage therapy, Phage manufacturing, Phage pharmacokinetics, Staphylococci, Periprosthetic joint infection

## Abstract

Phage therapy has recently attracted significant attention as a potential treatment for periprosthetic joint infections, yielding promising outcomes in several compassionate use cases. The absence of standardized treatment protocols is partly attributable to insufficient pharmacokinetic data regarding relevant phage administration routes and dosages. Another neglected aspect is the scalable manufacturing of pharmaceutical-grade phage preparations for preclinical testing. In this study, we address both challenges and present a scalable phage production process for the *Staphylococcus epidermidis*-specific phage COP-80B We prepared a highly purified phage suspension, as verified through qPCR, HPLC, NTA and short-read sequencing, which was used in a preclinical pharmacokinetic study in an uninfected mice model. Using a plaque assay, we determined phage concentrations in mouse organs over time after intraperitoneal and intra-articular application of 10^9^ phages. Intra-articularly administered phages persisted in the periarticular tissue for several days, entered the systemic circulation and were subsequently cleared from the liver and spleen. Conversely, intraperitoneally administered phages did not reach the intra-articular space. No adverse events and no changes in hematological parameters were observed in mice after phage application by either route, confirming the safety of a single-dose application. Our results emphasize the importance of local phage administration for sustained presence in periarticular tissue and provide valuable pharmacokinetic data to support the development of optimized treatment protocols for periprosthetic joint infections.

## Highlights

• Rapid and scalable production method yields pharmaceutical-grade phage preparation.
• Local phage administration results in sustained phage presence in the knee tissue.
• Systemic phage administration fails to deliver phages to the periarticular tissue.
• No observable toxic effects after phage COP-80B administration in mice.

## 1 Introduction

Bacterial infections remain an increasingly challenging problem in human medicine, particularly due to the development of antimicrobial resistance. Some conditions, such as bone and joint infections, are difficult to treat even in cases where resistance does not occur (Josse et al., 2019; Murray et al., 2022). Staphylococci are the most common pathogen in periprosthetic joint infections (PJI), capable of forming biofilm on implants resulting in infections that require treatment with a combination of surgery and antibiotic therapy (Osmon et al., 2013; Patel, 2023). Phage therapy has recently emerged as a promising complementary treatment for complicated PJI cases, mainly because phages are active against antimicrobial-resistant bacteria and have anti-biofilm activity (Kifelew et al., 2020; Song et al., 2021). Nevertheless, even a combined surgical-phage-antibiotic treatment approach is not always successful (Ferry et al., 2020; Munteanu et al., 2025; Ramirez-Sanchez et al., 2021). The success of phage therapy for PJI depends on the treatment protocols, which vary according to the clinical indication and usually involve the administration of single or sequential doses of phages administered intravenously, intra-articularly or both, requiring a personalized approach (Ferry, 2024). A deeper understanding of phage pharmacokinetics (PK) and clinical data is required to design more effective or even standardized treatment protocols.

Recent clinical studies confirmed the safety of phage therapy regardless of the route of administration. However, the effects of phage dose and route of administration on safety are not yet fully understood (Stacey et al., 2022). Preclinical studies generally report no apparent adverse events following phage administration, although increased spleen weight has been reported following high single or repeated doses (Tan et al., 2024; Wang et al., 2024). In addition, phages can induce both pro- and anti-inflammatory responses via up- or down-regulation of specific cytokines. Yet, it remains uncertain to what extent these responses are due to impurities in the phage preparations (Van Belleghem et al., 2017; Wang et al., 2024). The quality of phage preparations varies considerably between preclinical studies and product characterization is usually limited to the quantification of endotoxins, although other contaminants such as host cell DNA can also induce an immune response (Hemmi et al., 2000; Liu et al., 2021; Van Belleghem et al., 2017). Most recent preclinical studies have relied on phage preparations purified by cesium chloride density gradient ultracentrifugation and dialysis optionally coupled with cross-flow filtration for lysate concentration and affinity chromatography for endotoxin removal (Dhungana et al., 2021; Forti et al., 2018; Luong et al., 2020; Totten et al., 2022; Van Belleghem et al., 2017; Wang et al., 2024). While this approach results in relatively pure phage preparations, the main disadvantages of such production processes are poor scalability and long ultracentrifugation processing times. This makes chromatography-based downstream processes a useful alternative, especially as new column modalities are now available that enable more efficient endotoxin removal (Adriaenssens et al., 2012; Rebula et al., 2023).

In connection with the treatment of PJI, two studies were published in which the pharmacokinetics after intra-articular application of two staphylococcal phages were investigated. In the first study, *Staphylococcus-*specific phage K was administered either intravenously or intra-articularly at a dose of 10^11^ PFU in healthy and osteoarthritic rabbits. The intra-articularly administered phage was detected in the blood, but the intravenously administered phage was not detected in the synovial fluid (Totten et al., 2022). In the second study, the pharmacokinetics of *Staphylococcus epidermidis* phage vB_SepM-Alex was investigated in a rat model of periprosthetic joint infection after intra-articular application of 10^8^ PFU/animal, comparing PK parameters between uninfected and infected animals. Interestingly, the phage half-lives in the periarticular tissue were shorter in infected animals and the phage counts in the blood were below the detection limit, indicating poor systemic penetration from the intra-articular tissue (Young et al., 2024).

To fill existing knowledge gaps in scalable phage production and phage pharmacokinetics, we present small-scale production process together with the applied quality control, followed by a study on the distribution and elimination of the selected phage in the mouse model. As a model virus, we use the phage COP-80B, which has been shown to be strictly lytic and non-transducing, infects a broad spectrum of clinical strains, and has no genetic determinants for virulence factors, toxins, antibiotic resistance, or genes related to lysogenicity, making it a suitable candidate for phage product development (Štrancar et al., 2023).

## 2 Materials and methods

### 2.1 Bacterial strains and phage

*S. epidermidis* strain COB-Sec2 (GenBank accession number: JARGDW000000000, https://www.ncbi.nlm.nih.gov/nuccore/JARGDW000000000.1/) of commensal origin and free of prophages and toxin genes was selected as the phage propagation strain (Štrancar et al., 2023). The bacteria were grown in tryptic soy broth (TSB, Merck, Germany) or on agar plates (Biolife, Italy) at 37 °C.

The phage utilized in this study, COP-80B, is a lytic phage classified within the genus Sepunavirus. It has been previously isolated and characterized (Štrancar et al., 2023). The phage genome sequence is available in Genbank (accession number: OQ448194, https://www.ncbi.nlm.nih.gov/nuccore/OQ448194.2/). Phage quantification was performed either via double agar overlay plaque assay or drop plaque assay on TSB plates containing 1.2 % (w/v) agar and an overlay of 0.4 % (w/v) agar.

### 2.2 Phage cultivation and purification

Phage COP-80B was cultivated in a 2 L bioreactor (Solaris Biotech, Italy) equipped with three Rushton-type impellers and a medium volume of 1000 mL in liquid TSB medium supplemented with 10 mM MgSO_4_ (Sigma-Aldrich, Germany) and 1 mM CaCl_2_ (Fisher Chemical, USA). The bioreactor containing growth medium was sterilized at 121 °C for 30 min. To avoid excessive foam formation, 0.1 mL/L of antifoam AF204 (Sigma-Aldrich, Germany) was added aseptically. The medium was inoculated with 1 % (v/v) overnight culture of COB-Sec2 prepared in TSB medium and the culture was grown until the onset of mid-logarithmic growth at 37 ± 1 °C, pH 7.0 ± 1.0 and an airflow rate of 2 slpm. The pH was maintained via the automatic addition of sterile 15 % NH_4_OH (Sigma-Aldrich, Germany) or 18.5 % HCl (Merck, USA). The level of dissolved oxygen (DO) was monitored on-line and maintained above 20 % through automatic adjustment of the stirrer rate during the fermentation. The process was additionally monitored via offline optical density measurements. The culture was infected with phage COP-80B at a bacterial concentration of (4.0 ± 0.1) × 10^8^ CFU/mL and a multiplicity of infection (MOI) of 0.01. Following bacterial lysis, i.e. when the optical density dropped to the initial values, the lysate was centrifuged at 9000 g for 10 min. The supernatant was clarified through a capsular 0.8/0.45 µm pore size filter followed by a secondary filtration through a 0.45/0.22 µm pore size filter (Sartorius, Germany) to eliminate any remaining cell debris and medium components.

Two-step chromatography purification was performed using monolithic columns (Sartorius BIA Separations, Slovenia). Chromatography system ÄKTA Avant 150™ (Cytiva, USA) with UV signal at wavelengths of 260 nm and 280 nm was used. For the capture step, clarified lysate was adjusted to 1.7 M phosphate buffer and was loaded onto the CIMmultus® OH column (cat. no 911.8140–6), followed by a wash step with 1.7 M potassium phosphate buffer pH 7.0 and a linear gradient in 15 column volumes to 20 mM potassium phosphate buffer pH 7.0. For the polishing step, the OH elution fraction containing phages was diluted to decrease its conductivity to 18 mS/cm before being applied to the CIMmultus® QA column (cat. no 911.5113–6). The column was washed with 20 mM HEPES and 10 mM MgSO_4_ at pH 7.0 and elution was performed in a step gradient manner: initially to 20 mM HEPES, 10 mM MgSO_4_, and 0.6 M NaCl at pH 7.0, followed by 20 mM HEPES, 10 mM MgSO_4_, and 2 M NaCl at pH 7.0.

To concentrate and exchange the buffer of QA elution fraction, Pellicon XL 50 ultrafiltration cassette (Merck, Germany) was used. Buffer was exchanged 100 times against Dulbecco's Phosphate-Buffered Saline (DPBS) (Sigma-Aldrich, Germany) with 10 mM MgSO_4_ (Sigma-Aldrich, Germany) and the phage sample was concentrated 2 times.

### 2.3 In process control of phage titer, total protein and host-cell DNA

Phage titers (infectious phage numbers) were determined at each process step to determine process recovery with drop plaque assay on TSB agar plates, as was previously described in (Mazzocco et al., 2009). Briefly, sample serial dilution was prepared and 10 µL of each dilution was spotted onto the overlay agar of bacterial strain COB-Sec2. After overnight incubation at 37 °C, plaques in drops with 10–45 plaques were counted and phage titer (PFU/mL) was calculated.

The total protein content in samples was determined with the Micro BCA (µBCA) Protein Assay Kit (Thermo Fisher Scientific, USA) according to the manufacturer’s instructions.

The host cell DNA of the propagation strain COB-Sec2 was quantified using qPCR with primers previously published in the literature, with a slight modification in forward primer (PAN23S_mod_F: 5′ – CCATCGCTCAACGGATAAAAGC – 3′ and PAN23S_R: 5′ – GATGAGCCGACATCGAGGTGC – 3′) (Gaibani et al., 2013). Standard for the qPCR calibration curve was DNA of *S. epidermidis* strain COB-Sec2, which was extracted from liquid overnight culture using the High Pure PCR Template Preparation Kit (Roche, Switzerland) as described previously (Štrancar et al., 2023). Eluted DNA was quantified by Quant-iT PicoGreen dsDNA Reagent (Thermo Fisher Scientific, USA), aliquoted and stored at −20 °C for later use. A calibration curve was constructed based on tenfold serial dilutions of extracted DNA and the reaction efficiency of 85 % was determined (Ginzinger, 2002). The limit of quantification was 0.00011 ng/µL. The total volume of 20 µL qPCR reaction included: 10 µL of PowerUp SYBR Green Master Mix (Thermo Fisher Scientific, USA), 0.5 µL of each primer (Sigma-Aldrich, Germany), 8 µL of DEPC-treated water (Thermo Fisher Scientific, USA) and 1 µL of standard, sample or no template control. Samples were diluted in DEPC-treated water prior to analysis and no other pretreatment was performed. All the samples were analyzed in three different dilutions with two replicates per dilution. The qPCR was performed with QuantStudio 3 Real-Time PCR System (Thermo Fisher Scientific, USA) using the following thermal cycling conditions: UDG activation at 50 °C for 2 min, initial denaturation at 95 °C for 2 min followed by 40 cycles of 15 s at 95 °C and 1 min at 60 °C. The cycle ended with a dissociation curve at 95 °C for 15 s. Data was analyzed with QuantStudio Design and Analysis Software v1.5.1 (Thermo Fisher Scientific, USA).

### 2.4 Endotoxin determination

Measurements of endotoxins were performed on Endosafe® nexgen-PTS™ cartridge system (Charles River, USA) with Endosafe 0.05 EU/mL cartridges according to the manufacturer’s instructions. Samples were diluted in 1:1 dilution of LAL Reagent Water and Endotoxin-Specific Buffer (Charles River, USA).

### 2.5 SEC—HPLC analytical method

A PATfix® Model LPG system (Sartorius BIA Separations, Slovenia) equipped with a multiple-wavelength UV–VIS detector (190–700 nm, 8-channel deuterium lamp and 50 mm path length) and the software PATfix® 2.1 for data acquisition was used. A multi-angle scattering detector (MALS) Dawn Heleos-II (Wyatt Technology, USA) was integrated into the system. UV absorbance was monitored at wavelengths of 260 and 280 nm and light scattering was measured at an angle of 90°.

A TSKgel G5000PWXL (7.8 mm ID x 30.0 cm L) column (Tosoh Bioscience, Germany) was used with 50 mM potassium phosphate buffer with 300 mM NaCl, pH 7. Prior analysis, phage samples were diluted in the buffer either 10 times (phage lysate) or 4 times (purified COP-80B) and 200 µL of the diluted sample was injected. A flow rate of 0.5 mL/min was applied and the experiment was performed at 25 °C.

### 2.6 Nanoparticle tracking analysis (NTA)

Homogeneity of purified phage preparation was inferred from particle size distribution which was determined by Nanoparticle Tracking Analysis (NTA) using a NanoSight N300 (Malvern Instruments, United Kingdom) equipped with a blue laser module (488 nm). NTA software version 3.2 was used for the capture and analysis of the data. Phage samples were diluted in phosphate-buffered saline (PBS) to a working concentration resulting in 20–100 particles per video frame (10^7^–10^8^ PFU/mL). The samples were injected with a syringe pump into the device chamber and measurements were performed at room temperature. Each sample was recorded five times for 45 s at constant capture settings with manually adjusted camera level, focus and analysis parameters (detection threshold 4 or 5).

### 2.7 Transmission electron microscopy (TEM)

Formvar-coated grids were placed on drops of different phage suspensions for 5 min and negative staining was performed using 2 % phosphotungstic acid (PTA). The grids were examined using a transmission electron microscope JEOL JEM-1400 Plus (Tokyo, Japan) at 120 kV.

### 2.8 Genetic characterization of phage product

Phage DNA was extracted from purified phage suspension using the Phage DNA Isolation Kit (Norgen, Canada) according to the modified protocol from the manufacturer, as previously described (Štrancar et al., 2023). Sequencing libraries were prepared using the Nextera XTL library preparation kit (Illumina), and sequencing was performed using MiSeq or NovaSeq (Illumina) from a commercial vendor. Quality read trimming, read subsampling and genome assembly were performed as previously described (Štrancar et al., 2023). Unmapped reads were extracted from the *bam* file using samtools and assembled by Spades (Danecek et al., 2021). Obtained assemblies were analyzed by *blastn*^1^ against *S. epidermidis* plasmids (NCBI non-redundant database) and against the database containing the contigs belonging to the propagation host to identify contamination with non-target phage DNA. Next, a comparison of phage product and phage seed bank genome (available in Genbank, accession number: OQ448194) sequences was performed with FastANI to determine percent identity between the sequences (Jain et al., 2018). Variant detection in phage product was performed with Freebayes (Garrison and Marth, 2012).

### 2.9 Phage stability study

The stability of purified phage COP-80B was studied in three different final formulations (DPBS with 10 mM MgSO_4_, DPBS with 10 mM MgSO_4_ and 10 % (w/v) sucrose (Sigma-Aldrich, Germany) and DPBS with 10 mM MgSO_4_ and 15 % (v/v) glycerol (Sigma-Aldrich, Germany)) and on two different storage temperatures (4 °C and −80 °C), totaling in four different storage conditions. The average concentration of the four phage formulations at the start of stability study was (4.7 ± 0.5) × 10^11^ PFU/mL. Phage titer was determined over a period of 78 weeks by double agar overlay plaque assay with three technical replicates using bacterial lawn of phage propagation strain COB-Sec2, prepared from aliquots of the same bacterial working cell bank to prevent repeated freeze-thaw cycles (Kropinski et al., 2009).

### 2.10 Ethical approval

All experiments on animals were performed according to the directives of the EU 2010/63 and a protocol outlining the research described in this study was approved by the Administration of the Republic of Slovenia for Food Safety, Veterinary Sector and Plant Protection of the Ministry of Agriculture, Forestry and Foods, Republic of Slovenia (Permit Number U34401–3/2021/8).

### 2.11 Animals

Male C57BL/6_OlaHsd mice (8–10 weeks old) were purchased from Envigo, Italy. The animals were housed under a 12 h dark-light cycle with free access to water and maintenance chow (4RF21, gamma irradiation sterilized, Mucedola, Italy) in housing units (IVC system, Techniplast, Italy; up to 4 mice per cage) containing environmental enrichment (nesting materials and mouse tunnels). Animals were acclimatized a minimum of 2 weeks to daily handling before procedures. Animals were assigned to different groups with a randomization method using QuickCalcs (GraphPad).

### 2.12 Phage dosing and administration

Phage dose of 10^9^ PFU/animal (equals to (3–5) × 10^10^ PFU/kg body weight) was used and phages were applied as a single dose either intra-articularly or intraperitoneally. Phage solution concentrations for either application were calculated based on the phage dose and application volume; the solutions were prepared by dilution in sterile DPBS with 10 mM MgSO_4_.

Intra-articular (IA) application was performed under isoflurane anesthesia, and the right knee was shaved prior to application. Two µL of phage solution for IA were applied percutaneously with a 27 G needle and 10 µL syringe (Hamilton, USA) into the intra-articular space of the right knee. The volume of injection was determined based on the literature reporting that the 2 µL injection of methylene blue remains in the mouse intra-articular space without spreading (Carli et al., 2017). For intraperitoneal (IP) administration, 100 µL of the phage solution was injected into the lower right quadrant of the abdomen using a 27 G needle. Mice were gently restrained, and injections followed standard aseptic techniques. Post-injection, animals were monitored for any signs of distress. Control groups received DPBS with 10 mM MgSO_4_ (vehicle control solution).

### 2.13 PK study of cop-80b in vivo

Mice were divided into two groups, phage (*N* = 64) and vehicle control (*N* = 44) group. The phage group was further subdivided into mice receiving phages via IP (*N* = 32) or IA (*N* = 32) administration, respectively. The vehicle control group was subdivided into mice receiving the vehicle control solution via IP (*N* = 21) or IA (*N* = 23) route, respectively. Mice were euthanized at 0.25, 1, 2, 8, 24, 48, 72 and 96 h post administration (*N* = 4 per timepoint for the phage group and *N* = 2–4 per timepoint for the vehicle control group) (Fig. 3A).

### 2.14 Tissue harvest, processing and phage enumeration

At the end of the experiment, mice were weighed and euthanized by cervical dislocation, decapitation, and exsanguination into a 50 mL Falcon tube. An aliquot of 75 µL of blood was immediately collected separately into potassium EDTA-containing tubes (Sarstedt, Germany) for hematological analysis with Vetscan HM5 Hematology Analyzer (Zoetis, USA). The remaining blood was left at room temperature for 30 min to clot, after which serum was prepared according to standard protocol. After blood collection, organs (liver, spleen, kidneys, gonads, lungs, heart, brain, inguinal lymph node) were collected, weighed (liver, spleen, left kidney and heart), flash-frozen in liquid nitrogen and stored at −80 °C until processing. Lastly, periarticular tissues of the right limb were collected by the transverse cut of the tibia and femur near the margins of the joint capsule, leaving the capsule intact. The collected periarticular tissue was then flash-frozen in liquid nitrogen and stored in the same manners as the other organs. Due to the issues with blood sampling, we had to exclude two animals in IA group (one from phage group and one from vehicle control group) from hematology data analysis, the updated numbers of animals per group are available in the Table S5.

Tissue samples were weighed and homogenized in PBS with Precellys Minilys Tissue homogenizer (Bertin, France) using 2 mL Precellys CK14 soft tissue homogenizing kits (for liver, spleen, kidneys, gonads, lungs, heart, brain, inguinal lymph node) or MK28 hard tissue grinding kits (for periarticular tissue). The liver, spleen, kidneys, gonads, lungs, heart, brain and inguinal lymph node were homogenized at 4000 rpm for 4 × 20 s with 30‑*sec* pause in between cycles. Periarticular tissue was homogenized at 5000 rpm for 6 × 60 s with 60‑*sec* pauses on ice between cycles. The homogenates were then centrifuged at 5000 g for 1 min.

The serum and tissue homogenate supernatants were serially diluted in SM buffer and phage titers were determined by double agar overlay plaque assay as described previously (Kropinski et al., 2009). Briefly, 100 μL serial dilutions of serum or tissue homogenates were mixed with 100 μL of the bacterial strain COB-Sec2 and added to TSB overlay agar, which was poured onto TSB agar plates. After overnight incubation at 37 °C, plates with 20–200 plaques were counted, and phage titer in serum (PFU/mL), phage numbers per gram tissue (PFU/g tissue), or phage numbers per organ (PFU/organ) were calculated. The limit of quantification (LOQ) of the double agar overlay plaque assay was 500 PFU/mL as determined from titration of serial two-fold phage dilutions and evaluating titer linearity, precision and accuracy. The limit of detection (LOD) was 10 PFU/mL (i.e., 1 PFU per plate). LOQs for specific tissues were calculated as PFU/g tissue from phage titer LOQ (PFU/mL), average tissue sample mass (g) and homogenate volume. Phage titration was performed in duplicates.

To determine the recovery of infectious phages from mouse tissue samples, liver, serum and buffer (negative control) were spiked with phage COP-80B The spiked tissue samples were frozen, thawed, homogenized (liver) and serially diluted. Phage titer was determined as described above, and recovery was calculated as the phage titer of the spiked tissue sample divided by the phage titer of spiked buffer control. The experiment was performed independently two times with three replicates.

### 2.15 PK modeling

Phage numbers after intraperitoneal application in the liver and spleen and after intra-articular application in periarticular tissue were analyzed to obtain pharmacokinetic parameters with noncompartmental analysis with extravascular functionality using the PKSolver plug-in for Microsoft Excel (Microsoft, USA) (Zhang et al., 2010).

### 2.16 Data interpretation and statistical analysis

The results were visualized using Python software packages seaborn and matplotlib (Hunter, 2007; Waskom, 2021). Data are expressed as mean ± standard error of the mean (SEM) unless stated otherwise. Data normality was confirmed using Kolmogorov–Smirnov test. Comparisons were performed by a parametric test (unpaired *t*-test with Welch’s correction) using Prism 9.0.0 (GraphPad, USA). The Holm–Sidak correction method was used for multiple unpaired *t*-test comparisons. Differences with *P* < 0.01 were considered statistically significant.

## 3 Results

### 3.1 Phage propagation and purification

**Fig. 1:**
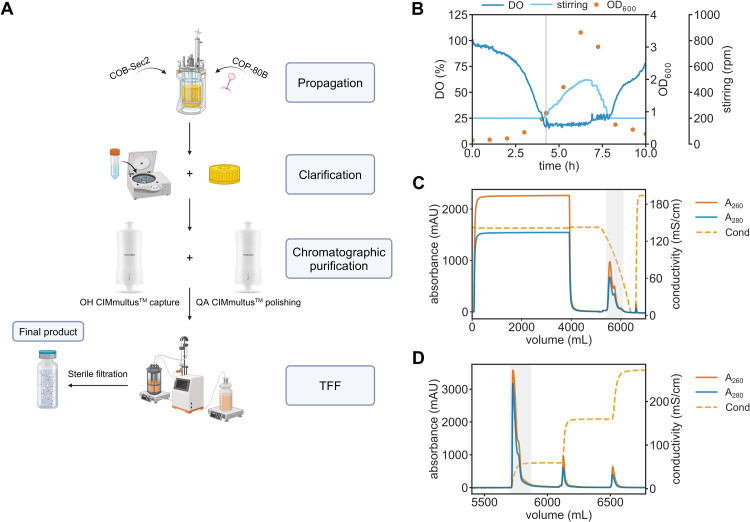
Phage COP-80B preparation process. (A) Schematic representation of phage preparation process. Created in BioRender. Str, V. (2025) https://BioRender.com/c59b936**(B)** Phage amplification process in stirred tank bioreactor. Dissolved oxygen (DO) was maintained above 20 % through automatic adjustment of the stirrer rate. Bacterial growth was monitored by offline optical density (OD_600_) measurements to determine the time point for phage infection (gray vertical line) and time point of population-wide bacterial lysis. **(C)** Phage particles from filtered cell lysates are captured on 80 mL CIMmultus OH monolithic column using hydrophobic interaction chromatography (HIC). Lysates were diluted to a 1.7 M final phosphate concentration in potassium phosphate buffer. Larger particles are retained on the column and eluted in the descending phosphate concentration. **(D)** Elution profile of COP-80B polishing step. Phage-containing elution fraction after HIC capture step is further polished using anionic chromatography (AEX) on 80 mL CIMmultus QA monolithic column. To reduce the conductivity of the sample, phage elution was diluted in 20 mM HEPES, 10 mM MgSO_4_, pH 7.0. Phages were eluted in a step gradient to a buffer containing 0.6 M NaCl. Detection: UV at 260 nm (orange) and 280 nm (blue), conductivity (yellow, hatched).

A single batch of the phage COP-80B was prepared for the pharmacokinetic study according to the procedure shown in Fig. 1A. The phage was propagated in a liquid culture of COB-Sec2 in a stirred tank bioreactor in a batch process that yielded a phage lysate with titers of 8 × 10^10^ PFU/mL (Fig. 1B). The combination of infection in the early exponential growth phase and low MOI resulted in a 10-hour process that ended with lysis of the bacterial culture and subsequent harvest. Lysate clarification was performed by centrifugation and microfiltration, followed by two monolith convective interaction media (CIM) chromatography steps. In the first chromatography step, phages and other impurities with larger molecular weight bound to the CIMmultus OH column in hydrophobic interaction chromatography mode (HIC) and were eluted in a gradient to low-salt buffer (Fig. 1C). In this step, we achieved a 96 % reduction in the amount of host cell DNA (hcDNA) (Table 1). In addition, the amount of total proteins determined with the µBCA assay was reduced by at least 99 % without a simultaneous decrease in the number of infectious phages (Table 1). The eluted fraction from the OH column was further purified by anion exchange chromatography (AEX). The bound phages were eluted in a step gradient to a buffer containing 0.6 M NaCl (Fig. 1D). In this step, we achieved additional removal of hcDNA, obtaining a cumulative reduction of 99.9 % (Table 1). The recovery of infectious phages in the second chromatography step was 75 % (Table 1). Finally, we used an ultrafiltration cassette to concentrate the phage suspension and exchange the high salt buffer (0.6 M NaCl) for DPBS with 10 mM MgSO_4_. The overall recovery of phages was 60 %, with a total protein depletion of 99 % and a removal of hcDNA of >99 % (Table 1). Phage preparation was sterile-filtered, and its sterility was confirmed by an in-house sterility testing protocol.

**Table 1:** Phage titer recovery, total protein levels, host cell DNA levels and endotoxin levels in different process steps.

Sample	Total titer (Spot assay)		Total proteins (µBCA assay)		Host cell DNA (qPCR assay)		Total endotoxins (Endosafe)	
	Total PFU	Total PFU yield ( %)	mg	Depletion ( %)	ng	Depletion ( %)	EU/mL	EU/10^10^ PFU
Filtered phage lysate	1.3 × 10^14^	100	17,145		5.1 × 10^6^		0.671	0.09
OH elution	1.3 × 10^14^	> 100	213	98.8	1.9 × 10^5^	96.4	NA	NA
QA elution	9.5 × 10^13^	75	200	98.8	<19	>99.9	1.38	0.03
Final product	7.6 × 10^13^	60	155	99.1	<8	>99.9	1.84	0.02

PFU: plaque forming unit; EU: endotoxin unit; NA: not analyzed.

### 3.2 Quality control of the phage suspension for PK study and its stability

**Fig. 2:**
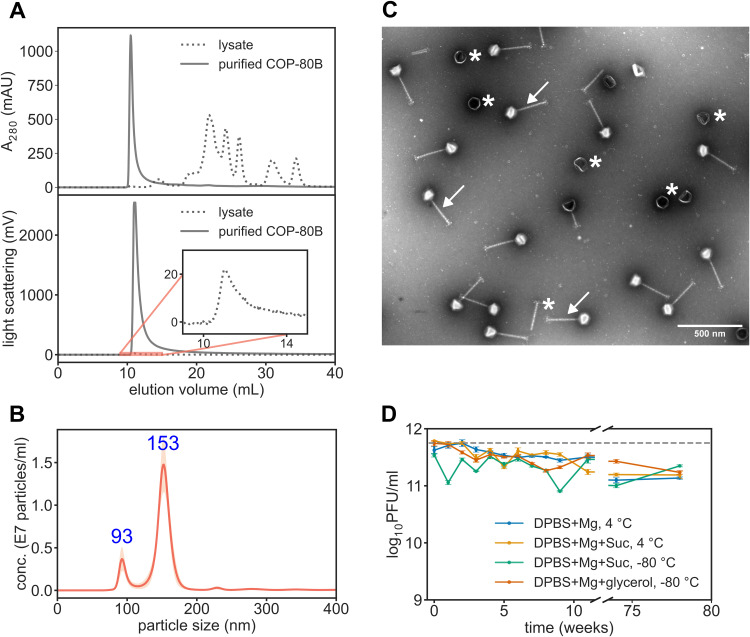
Characterization of the purified phage COP-80B (A) HPLC-SEC using absorbance and MALS detectors is used for comparing purity of COP-80B lysate and purified COP-80B Purified COP-80B lacks impurities detected with absorbance detector at higher elution volumes. **(B)** NTA size distribution of purified COP-80B with mean for both peaks. Errors in shaded area represent SEM (*n* = 5). **(C)** Transmission electron micrograph of purified COP-80B, where intact particles (white arrows) and disintegrated particles are seen (heads without tails, tails only; white asterisks). Scale bar is 500 nm. **(D)** Stability of purified COP-80B over 78 weeks: repeated phage titers in different formulations stored on 4 °C and −80 °C. Initial formulation titer is plotted as a hatched asymptote; values are plotted as means with SEM (*n* = 3). conc.: concentration; PFU: plaque-forming unit; Mg: magnesium sulphate; Suc: sucrose.

The removal of protein impurities was demonstrated using the HPLC-SEC method on a system equipped with an inline MALS detector. The phage samples were injected onto a size exclusion chromatography (SEC) resin that excludes phages from entering the bead pores, separating them from lower molecular weight contaminants, resulting in phage elution in the void volume. The absorbance chromatogram of the phage lysate shows a small peak at 11 mL and multiple peaks between 13 mL and 38 mL, while the light-scattering signal shows only one small peak at 11 mL, indicating elution of larger particles, including phages, at 11 mL (Fig. 2A). The chromatogram of purified phage shows a single peak at 11 mL for absorbance and light-scattering channels (Fig. 2A). Comparing chromatogram profiles of lysate and the final COP-80B suspension, it is apparent that the two-step chromatography purification efficiently removed the low molecular weight impurities.

Endotoxin presence was monitored despite the Gram-positive propagation host being used to detect potential contamination during the purification process. We determined that the endotoxin levels were low throughout the whole production process and the final product endotoxin content was below the regulatory limit of 5 EU/kg*h (Table 1).

According to the nanoparticle tracking analysis (NTA), the phage product displayed a fairly uniform distribution, with phage particles averaging 153 nm in size; however, a notable peak of particles measuring 93 nm is also observed (Fig. 2B). We hypothesize this peak represents tail-less phage heads, which have an average radius of 95 nm, as measured from transmission electron micrographs (Fig. 2C). In addition to tail-less heads, phage tails without heads are also observed on the micrographs, indicating the presence of degraded phages, which represent an important product-related impurity in our phage product. From NTA peak areas we estimated the ratio of intact versus degraded phage particles to be 6:1.

Another aspect of phage product quality is absence of non-target phage DNA, and similarity with phage seed bank. We evaluated this with short-read sequencing, confirming the identity of the phage product to be COP-80B Comparative analysis with phage seed bank sequence showed 99.99 % identity. One single nucleotide polymorphism (SNP) was identified in the gene product 80B_00043, which is annotated as hypothetical protein, where threonine (polar amino acid) in phage seed bank is replaced by isoleucine (hydrophobic amino acid) in phage product (Table S1).

Furthermore, we determined the stability of the phage product over 78 weeks in different formulations of DPBS buffer with 10 mM MgSO_4_ at two regularly used storage temperatures, i.e. 4 °C and −80 °C. We have shown that phage titer gradually decreases for all tested conditions and that on average the phage titer drops for -(3.1 ± 0.7) × 10^11^ PFU/mL until week 78 (Fig. 2D). Addition of 10 % (w/v) sucrose does not improve long-term stability at 4 °C and sucrose is not a better cryopreservative than glycerol for storage at −80 °C (Table S2). We have observed considerable variations in titer of formulation with sucrose at −80 °C overtime (Fig. 2D).

To sum up, we have produced phage COP-80B preparation, which is 99.99 % identical to its phage seed bank and has minimal presence of non-phage DNA. Quantification of endotoxins and host cell DNA in the preparation revealed that each phage-injected mouse in the study received approximately 0.002 endotoxin units, which is well below the regulatory limit of 0.1 EU/dose for a 20 g mouse (5 EU/kg*h, https://www.fda.gov/inspections-compliance-enforcement-and-criminal-investigations/inspection-technical-guides/bacterial-endotoxinspyrogens), and <0.2 pg of hcDNA, which is below the regulatory limit of 10 ng/dose (Yang, 2013).

### 3.3 Pharmacokinetic study

In the preclinical mouse model, the plaque assay was used as the main analytical method to enumerate infectious phages. To test whether the tissue homogenate-derived compounds or homogenization process affect phage infectivity, we determined the recovery of the spiked phage in the selected tissues. The recovery of phage COP-80B from serum was 102 ± 14 % and 125 ± 11 % for liver tissue, confirming that neither tissues themselves nor tissue homogenization processing affected phage infectivity.

**Fig. 3:**
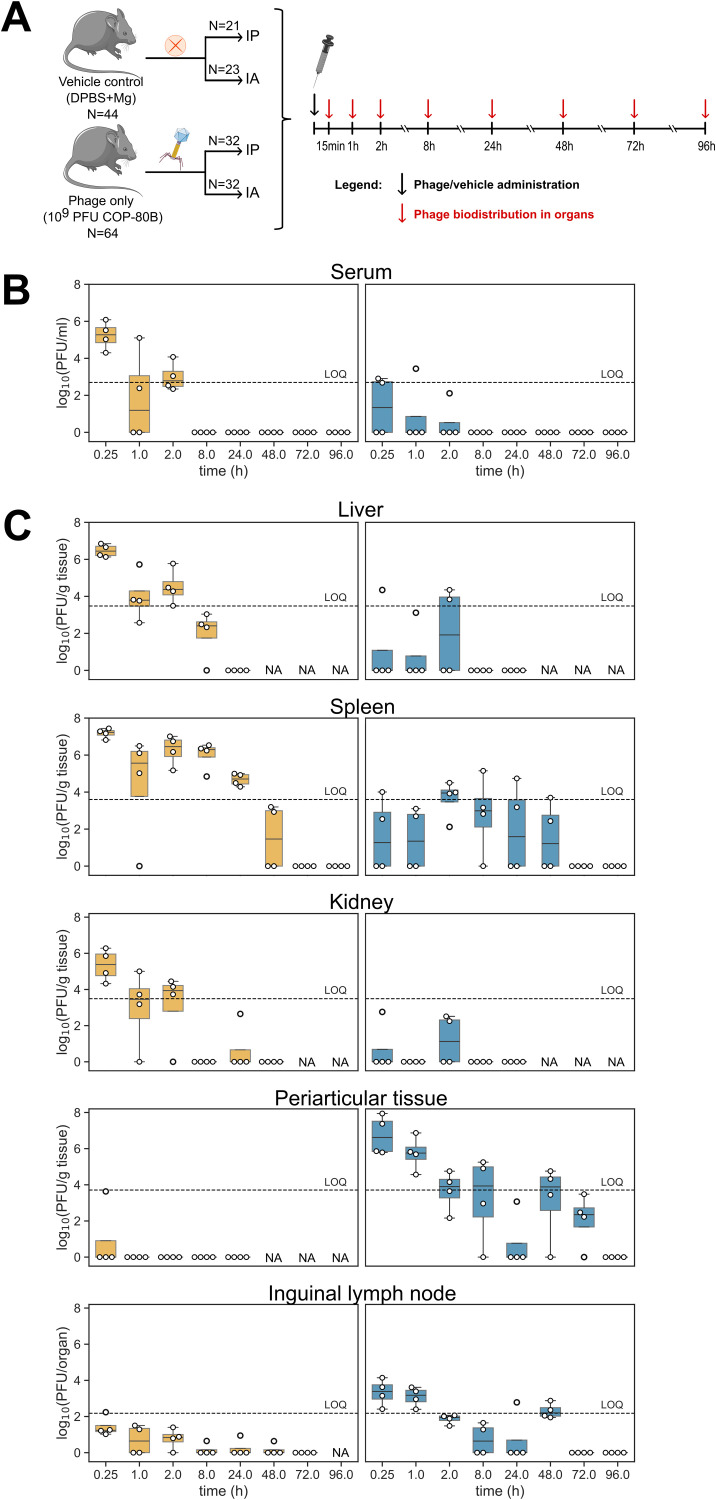
Pharmacokinetics and biodistribution of phage COP-80B following intraperitoneal (IP) and intraarticular (IA) administration in C57BL/6_OlaHsd mice. (A) Schematic representation of experimental design. Phage kinetics in serum and phage biodistribution in organs were performed following COP-80B IP and IA administration in mice. Mice were allocated into groups of four (receiving phage) and two to four (receiving vehicle control). (B) Kinetics of infectious COP-80B phages in serum following IP (yellow) and IA (blue) administration at a dosage of 10^9^ PFU in healthy C57BL/6_OlaHsd mice. Phage titer was obtained by double agar overlay plaque assay, phage titer is expressed as plaque-forming units (PFU) per milliliter in serum, and the limit of quantification (LOQ) is 2.7 log_10_PFU/mL. (C) Biodistribution of infectious phages in organs (liver, spleen, kidney, periarticular tissue of the right knee and inguinal lymph node of the injected (right) leg) following a single IP (yellow) and IA (blue) administration in healthy mice. Active phage titer is expressed as PFU per gram of each organ or PFU per organ (for inguinal lymph node); the LOQ for liver is 3.5 log_10_PFU/g tissue, LOQ for spleen 3.6 log_10_PFU/g tissue, LOQ for kidney 3.5 log_10_PFU/g tissue, LOQ for periarticular tissue 3.7 log_10_PFU/g tissue and the LOQ for lymph node is 2.2 log_10_PFU/organ. Phage titer was determined by double agar overlay plaque assay, and values are plotted as boxplot with the box extending from the first quartile (Q1) to the third quartile (Q3) of the data, with a line at the median. The whiskers extend from the box to the farthest data point lying within 1.5x the inter-quartile range (IQR) from the box. Flier points are those past the end of the whiskers (*N* = 4). NA: not analyzed, PFU: plaque-forming unit.

After IP application, phages could be detected and quantified in mouse serum 0.25, 1 and 2 h post-administration, reaching maximum concentration at 0.25 h (median of 1.9 × 10^5^ PFU/mL) (Fig. 3B). Generally, IP applied phages reached maximum tissue concentrations at 0.25 h post-administration and their concentration gradually decreased by 72 h post-administration when all phages were cleared (Fig. 3C). IP applied phages could be detected in liver at 0.25, 1, 2 and 8 h after administration and maximum concentration was determined at 0.25 h (median of 2.8 × 10^6^ PFU/g tissue); similarly, phages were detected in the spleen until 48 h after application with maximum concentration determined at 0.25 h (median of 1.7 × 10^7^ PFU/g tissue). Phages were detected also in kidneys at 0.25, 1, 2 and 24 h after IP administration with maximum concentration determined at 0.25 h post-administration (median of 2.4 × 10^5^ PFU/g tissue). Phage presence in periarticular tissue after IP application was confirmed in only one animal (*N* = 1) at 0.25 h post-administration, with a concentration of 4.3 × 10^3^ PFU/g tissue, suggesting poor penetration of phages from serum to periarticular tissue. However, IP-administered phages were also detected in the inguinal lymph node for up to 48 h in individual animals, although the phage levels remained below the quantification limit (Fig. 3C).

IA-administered phages were detected in the periarticular tissue for up to 72 h, with the highest concentration detected at 0.25 h (median of 4.2 × 10^6^ PFU/g tissue), indicating a long persistence at the target site of administration. In serum, phages could be detected only in 4 out of 12 animals at 0.25, 1 and 2 h after IA administration. IA-applied phages were detected in the liver, spleen and kidney only in individual animals for up to 2 h (liver and kidney) or 48 h (spleen) post-administration with phage concentrations generally below the quantification limit (Fig. 3C). Phages were also detected and quantified in the inguinal lymph node of phage-injected leg, reaching a maximum number of 2.4 × 10^3^ PFU at 0.15 h and then gradually decreasing to undetectable levels at 72 h.

Phages were detected also in other organs, especially after IP application. In heart, lungs and gonads, phages were detected for up to 8 h post-administration with the highest concentration determined at 0.25 h post-administration (Fig. S1). Similarly, phages were detected in the brain for up to 2 h post-administration. Phages, applied intra-articularly, were detected in the heart, lung, brain and in gonads but only in individual animals, in lower numbers (phage numbers were below quantification limit) and only at the earliest time points (Fig. S1). We have not detected phages in any tissue from mice in the vehicle control groups.

Importantly, no adverse effects were observed in mice following phage administration. The general well-being of the animals was monitored daily by assessing their behavior, including activity levels, feed and water intake, weight gain, and the appearance of the injection site. Pain assessment was conducted using the Mouse Grimace Scale (MGS), which evaluates facial expressions such as orbital tightening, nose and cheek bulging, and changes in ear and whisker position (Langford et al., 2010). No signs of health deterioration appeared, and no animals reached humane endpoints. To assess the impact of phage application on body mass, animals were weighed before euthanasia. No significant differences (*P* < 0.01) were observed in body weight or liver, spleen, kidney, or heart weight relative to body weight between phage-treated groups and vehicle control groups administered via IP or IA injection (Table S3). These findings indicate that phage administration had no significant impact on the overall well-being of the animals.

Furthermore, hematology data comparison showed similar values for analyzed parameters in phage and vehicle control groups regardless of application route with two exceptions (Table S4 and S5). A significant difference was observed in platelet count between groups receiving IP phage and vehicle control only at one time point, 2 h post-administration. Additionally, mean corpuscular hemoglobin values were significantly different between groups receiving phage and vehicle control intra-articularly also only at a single time point (24 h post-administration). As no consistent trends were observed, these differences are considered incidental.

To evaluate pharmacokinetics in different tissues, we performed noncompartmental pharmacokinetic analysis. For IA application, only analysis of the detected phages in periarticular tissue was performed, because the concentrations in other tissues were mostly below LOQ. The estimated half-life in periarticular tissue is 12.19 ± 7.07 h and the maximum concentration was reached within 0.25 h. The estimated mean residence time (MRT) was 10.81 ± 9.37 h; other parameters are presented in Table 2. For IP application, we could determine the parameters only for the spleen and partly for the liver; phage titers in serum decreased below the LOQ too quickly and therefore parameters could not be calculated. For the spleen, the estimated half-life was 5.17 ± 1.76 h and the estimated mean residence time (MRT) of the phages was 4.41 ± 1.00 h. Other calculated parameters are presented in Table 3. For the liver, complete results are presented in Table S6.

**Table 2:** Pharmacokinetic parameters of phage COP-80B in periarticular tissue following intra-articular application (10^9^ PFU).

Parameter	Unit	average ± SEM
λ_z_	1/h	1.18 ± 1.06
t_1/2_	h	12.19 ± 7.07
t_max_	h	0.25 ± 0.00
C_max_	PFU/(g tissue)	2.82 × 10⁷ ± 2.05 × 10⁷
AUC _0-t_	PFU/(g tissue)*h	1.83 × 10⁷ ± 0.98 × 10⁷
AUC_0–∞_	PFU/(g tissue)*h	1.92 × 10⁷ ± 0.96 × 10⁷
AUMC_0–∞_	PFU/(g tissue)*h^2^	1.03 × 10^8^ ± 0.75 × 10^8^
MRT_0–∞_	h	10.81 ± 9.37
V_z_/F_obs_	(PFU)/(PFU/(g tissue))	170.47 ± 122.85

λ_z_: terminal elimination rate constant estimate; t_1/2_: half-life estimate; t_max_: time of maximum concentration; C_max_: maximum concentration estimate; AUC_0–t_: Area under the concentration-time curve (total exposure estimate) from zero to the to last measurable concentration; AUC_0–∞_: area under concentration-time curve (total exposure estimate) over time; AUMC_0–∞_: area under the moment curve extrapolated to infinity; MRT_0–∞_: estimate of mean residence time extrapolated to infinity; V_z_/F_obs_: apparent volume of distribution.

**Table 3:** Pharmacokinetic parameters of phage COP-80B in spleen following intraperitoneal application (10^9^ PFU).

Parameter	Unit	average ± SEM
λ_z_	1/h	0.18 ± 0.04
t_1/2_	h	5.17 ± 1.76
t_max_	h	0.25 ± 0.00
C_max_	PFU/(g tissue)	1.71 × 10⁷ ± 0.44 × 10⁷
AUC _0-t_	PFU/(g tissue)*h	4.68 × 10⁷ ± 1.16 × 10⁷
AUC_0–∞_	PFU/(g tissue)*h	4.72 × 10⁷ ± 1.16 × 10⁷
AUMC_0–∞_	PFU/(g tissue)*h^2^	2.23 × 10^8^ ± 0.70 × 10^8^
MRT_0–∞_	h	4.41 ± 1.00
V_z_/F_obs_	(PFU)/(PFU/(g tissue))	27.45 ± 17.80

λ_z_: terminal elimination rate constant estimate; t_1/2_: half-life estimate; t_max_: time of maximum concentration; C_max_: maximum concentration estimate; AUC_0–t_: Area under the concentration-time curve (total exposure estimate) from zero to the to last measurable concentration; AUC_0–∞_: area under concentration-time curve (total exposure estimate) over time; AUMC_0–∞_: area under the moment curve extrapolated to infinity; MRT_0–∞_: estimate of mean residence time extrapolated to infinity; V_z_/F_obs_: apparent volume of distribution.

## 4 Discussion

This study addresses the gap in knowledge regarding scalable production and characterization of therapeutic-grade phages for preclinical use. We prepared a high-purity phage preparation of *S. epidermidis* phage COP-80B and evaluated its safety and pharmacokinetics in a mouse model following single-dose administration via intraperitoneal (IP) and intra-articular (IA) routes.

The production process involved batch mode propagation in a bioreactor followed by purification by monolith liquid chromatography (Fig. 1A). This production setup enables scalability, cGMP compliance and potential transferability to other phages, resulting in a high-titer formulation with low levels of process-related impurities. Compared to ultracentrifugation-based purification protocols, monolith chromatography-based purification is more time-efficient and avoids the use of potentially problematic chemicals, such as cesium chloride, chloroform, or Triton X-100 (Luong et al., 2020). However, transferability to other phages, particularly those with Gram-negative hosts, will need to be explored further, as it was shown that the QA polishing step might not be as effective for endotoxin removal as other, newer, column modalities (Rebula et al., 2023). To address characteristics of phage preparations, which are often underreported, we have performed extensive product characterization including quantification of hcDNA and endotoxins, estimation of host cell protein reduction and estimation of product-related impurities (Liu et al., 2021).

We report a substantial decrease in total protein levels in the final phage product compared to the phage lysate, with HPLC-SEC results indicating effective removal of lower molecular weight impurities. However, we did not quantify the concentration of host cell proteins in the final product as we lacked a specific method to detect these contaminants (Table 1, Fig. 2A). To our knowledge, there are currently no established methods to quantify host cell proteins in phage-based products. Two methods that are already in use in gene therapy viral vector and biologics manufacturing are commercially available ELISA kits or liquid chromatography coupled with tandem mass spectrometry (LC-MS/MS). The latter is potentially more useful for quality control of phage products as commercial ELISA kits are only available for a small number of bacterial hosts (Pilely et al., 2022).

From the perspective of the human immune system, phages are proteinaceous macromolecular structures, eliciting innate and humoral immune responses like any other virus (Van Belleghem et al., 2018). In this respect, phages are similar to other viral-based therapeutics. Current standards for several viral vectors demand quantification of total particle numbers relative to the infectious viruses, to estimate the presence of product-related impurities (Wunderli et al., 2020). Phage-related product impurities, mainly degraded phage particles (empty heads, tail-less heads, fragmented phage tails) and phage aggregates, have been characterized with various methods such as dynamic light scattering, TEM, NTA and interferometric light microscopy (Dharmaraj et al., 2023; Sausset et al., 2023). Analogous to viral vector-based therapeutics, degraded phage particles could contribute to the overall viral load, increasing phage protein-triggered immune response, potentially impacting treatment efficacy in prolonged therapies (McColl-Carboni et al., 2024; Suh et al., 2022). To address the presence of product-related impurities in our final product, we performed NTA and TEM, with NTA being regarded as the most precise method for size distribution determination (Sausset et al., 2023). Whereas we did not observe phage aggregation, our estimate is that at least one in seven COP-80B phage capsids is tail-less, indicating that the upstream and downstream processes should be optimized for lower generation of defective particles and more efficient removal of the degraded phages (Fig. 2B, 2C).

Phage suspension stability is important to ensure phage efficacy is not compromised as has happened in one of the clinical studies (Jault et al., 2019). It has been demonstrated that phage lysates are generally stable at 4 °C for extended time periods, however less data is available for purified preparations (Ackermann et al., 2004). DPBS buffer has been shown as a suitable formulation buffer for storage of highly concentrated purified stocks at 4 °C for longer periods of time, however a titer drop of 1 log_10_PFU/mL is usually observed (Duyvejonck et al., 2021). To enhance long-term stability, we wanted to test sucrose, as it is often used as a stabilizer and cryopreservative in liquid preparations of adenoviruses (Pelliccia et al., 2016). We have shown that sucrose does not significantly enhance COP-80B phage stability at 4 ° nor −80 °C, compared to glycerol (at −80 °C) or the buffer alone (at 4 °C) (Fig. 2D).

Regulatory standards for phage preparations for therapeutic use are still under development. Monitoring and reporting levels of endotoxin and host cell DNA, key impurities that can induce an innate immune response, is essential in phage preparations for preclinical safety studies to prevent drawing incorrect conclusions (Chow et al., 2021; Hemmi et al., 2000; Kumar et al., 2009; Raetz and Whitfield, 2002; Wang et al., 2024). In the recent case series on phage therapy for orthopedic infections, it was reported that impurities in phage preparations caused local inflammation. However, the use of suitably purified phages that were prepared using the purification methodology described in this paper, caused no apparent adverse effects, which we attribute to their exceedingly high purity (Munteanu et al., 2025). We show that the phage product produced and used in this study meets the stringent regulatory criteria regarding endotoxin and host cell DNA content.

This study evaluates the pharmacokinetics and biodistribution of the *S. epidermidis* phage COP-80B following a single therapeutically relevant dose in mice, comparing intra-articular and intraperitoneal administration routes. While systemic (IP) and local (IA) routes can be combined with repeated dosing to enhance therapeutic efficacy, we focused on single-dose kinetics to establish baseline parameters for this phage strain (Ferry, 2024). Our decision was motivated by the lack of pharmacokinetic data on alternative routes of administration, such as intra-articular application, which is particularly relevant for the treatment of PJI (Suh et al., 2022). Local (i.e. intra-articular) application is considered more appropriate as it ensures a high concentration of phages at the site of infection. Systemic application may still be useful as it allows phage delivery to locations not reached by local application and may be better in case of repeated administration, especially when repeated local administration is contraindicated (Ferry, 2024). Furthermore, we examined phage clearance from mouse organs, as this phage-strain-specific trait impacts the persistence of active phages in the body, thereby affecting their antibacterial effectiveness (Dąbrowska, 2019; Oliveira et al., 2009). Finally, we aimed to perform a primary safety assessment by comparing hematological parameters and relative organ weights between phage-administered mice and control animals, receiving vehicle control solution.

After IP application, we observed a rapid decline in serum phage numbers, with the serum titer being 0.0003 times the hypothetical value calculated from the phage dose and its dilution in blood volume at 15 min post-administration (Fig. 3B). Since no titer reduction was observed during sample processing, we attribute this rapid decline to immediate in vivo phage capture, filtration and neutralization after systemic application, as was previously observed and reviewed (Dąbrowska, 2019). Phage numbers after IP administration were highest in the liver and spleen already after 15 min post-administration and phage retention was the longest in the spleen (Fig. 3C), which is consistent with previous findings involving various phages in different animal models (Inchley, 1969; Smith and Huggins, 1982; Uhr and Weissmann, 1965). Phages were detected in serum after IA application only in some animals and in much lower concentrations, with similar trends observed in the liver and spleen. This is in accordance with the previously published data and suggests that local application minimizes systemic exposure (Albac et al., 2020; Totten et al., 2022). Phage presence after IP application in periarticular tissue was confirmed only in a single animal at 0.25 h post-administration suggesting poor phage delivery from the systemic circulation – this is consistent with findings from a rabbit model (Totten et al., 2022). Meanwhile, phage concentrations after IA application remained detectable in periarticular tissue even after 72 h and phages were also detected in inguinal lymph nodes for up to 48 h. Previous studies have shown that phages accumulate in lymph nodes following intraperitoneal and subcutaneous administration (Hodyra-Stefaniak et al., 2015; Wottlin et al., 2022). This accumulation is thought to occur because lymph nodes act as filters, trapping larger particles like phages from the lymphatic fluid before it reaches the systemic circulation (Gretz et al., 2000; Rantakari et al., 2015). We observed no differences in animal behavior, animal weight, relative organ weight or hematological parameters in phage versus vehicle control groups, which is in accordance with already reported data suggesting no major toxic events occur after phage application (Tan et al., 2024; Totten et al., 2022; Wang et al., 2024). These findings underscore the advantage of intra-articular administration for achieving sustained local phage presence while minimizing systemic exposure, supporting its potential relevance for targeted treatment of periprosthetic joint infections.

Pharmacokinetic parameters for serum and most tissues could not be calculated because the low number of infectious particles could not be reliably detected with plaque assay. Although qPCR is a more sensitive method, it does not distinguish between infectious and non-infectious particles (Totten et al., 2022). Therefore, the use of qPCR would limit the applicability of pharmacokinetic parameters, which are most relevant when based on viable phage titers. In addition, the distribution of sampling time points in our study also limited the accurate determination of the PK parameters. To accurately calculate these parameters, additional sampling time points within the first 24 h after administration would be beneficial, as phage levels in serum decline rapidly during this period. After intra-articular administration, the phage COP-80B showed a higher terminal elimination constant, a longer half-life and a longer mean residence time in the periarticular tissue than the *S. epidermidis* phage vB_SepM_Alex (tested at 10^8^ PFU/animal in a rat model) (Young et al., 2024). This could be due to the higher phage dose used in our study (10^9^ PFU/animal). However, the considerable variability of parameters in both studies emphasizes the need for further studies with more animals per time point and optimizing the distribution of sampling times to ensure more accurate determination of PK parameters.

An important limitation of our study was the exclusive use of healthy, non-infected animals. Testing on the infected animals would provide additional value, since phages can self-replicate at the site of the infection, although recently published findings suggest no significant increase in phage numbers in periarticular tissue due to the knee infection (Young et al., 2024). We opted to test a single dose, selecting it based on published case reports using intra-articular doses ranging from 10^9^ to 10^11^ PFU (Doub et al., 2022, 2021, 2020; Ferry et al., 2021). The dose tested in our study would correspond to approximately 3 × 10^12^ PFU per human, which is substantially higher than the doses currently employed in compassionate use cases. We hypothesize that higher doses may enhance efficacy; however, this assumption requires further validation. Testing several phage dosages would be reasonable to gain a better understanding of their effect on pharmacokinetics and biodistribution, and to determine the optimal dosage for clinical efficacy, however this remains to be determined in further studies. Mice were chosen as the experimental model because a large number of animals (*N* = 108) was required for the determination of phage biodistribution at eight different time points – a scale that would be difficult to achieve with larger or more complex animal models. A potential limitation of the selected model was the difficulty in accurately quantifying phages at the site of local administration. Due to the small knee size, synovial fluid sampling was not feasible; therefore, we opted to harvest the knee capsule along with surrounding tissues. Another limitation is related to the imprecision of intra-articular application of a small volume (2 µL), which might result in variability of the determined phage numbers in the intra-articularly injected group, influencing variability of the calculated pharmacokinetic parameters.

In summary, we describe the preparation of a highly purified phage suspension of the *S. epidermidis*-specific phage COP-80B, which was used to study the phage pharmacokinetics in mice. We have shown that intraperitoneal administration is not sufficient to deliver phages to the periarticular tissue in healthy mice. Conversely, intra-articular administration ensures the persistence of infectious phages in the periarticular tissue, confirming its relevance for the treatment of prosthetic joint infections (PJI). The local administration also minimizes systemic exposure to phages, which could suppress the development of phage-neutralization antibodies. Our study offers important insights into phage pharmacokinetics and safety, supporting the need for future research involving varied dosing strategies and infection models to guide clinical translation.

## Funding

This work was supported by Slovenian Research Agency with grants no L3–2620, J7–2603, and P3–0387. The funders had no role in study design, data collection and interpretation, or the decision to submit the work for publication.

All authors attest they meet the ICMJE criteria for authorship.

## CRediT authorship contribution statement

**Vida Štilec:** Writing – review & editing, Writing – original draft, Methodology, Investigation. **Monika Marušić:** Investigation. **Nika Janež:** Writing – review & editing, Methodology, Investigation, Conceptualization. **Urban Bezeljak:** Writing – review & editing, Methodology, Investigation. **Lucija Rebula:** Writing – review & editing, Methodology, Investigation. **Maja Leskovec:** Writing – review & editing, Methodology, Investigation. **Rihard Trebše:** Writing – review & editing. **Simon Horvat:** Writing – review & editing, Resources, Methodology, Investigation, Conceptualization. **Matjaž Peterka:** Writing – review & editing, Writing – original draft, Supervision, Resources, Methodology, Funding acquisition, Conceptualization.

## Declaration of competing interest

The authors declare the following financial interests/personal relationships which may be considered as potential competing interests:

Lucija Rebula, Maja Leskovec report chromatographic columns were provided by Sartorius BIA Separations d.o.o. Lucija Rebula reports a relationship with Sartorius BIA Separations d.o.o. that includes: employment. Maja Leskovec reports a relationship with Sartorius BIA Separations d.o.o. that includes: employment. If there are other authors, they declare that they have no known competing financial interests or personal relationships that could have appeared to influence the work reported in this paper.

## Data Availability

Data will be made available on request.

## Appendix Supplementary materials

Image, application 1

Image, application 2

## Footnotes

1 https://blast.ncbi.nlm.nih.gov/Blast.cgi
