# Supplementary material for: Preparation and pharmacokinetic evaluation of Staphylococcus phage COP-80B for treatment of periprosthetic joint infections in a mouse model
Source: Virus Res. 2025 May 31;357:199592. doi: 10.1016/j.virusres.2025.199592 (PMC12173072; doi:10.1016/j.virusres.2025.199592)
Supplement: Supplementary file 1 [file mmc1.pdf]

Preparation and pharmacokinetic evaluation of *Staphylococcus* phage COP-80B for treatment of periprosthetic joint infections in a mouse model

Vida Štilec[1,2,#], Monika Marušić[1], Nika Janež[1], Urban Bezeljak[1,3], Lucija Rebula[4], Maja Leskovec[4], Rihard Trebše[5,6], Simon Horvat[2], Matjaž Peterka[1]

Affiliations

[1] COBIK, Mirce 21, 5270 Ajdovščina, Slovenia

[2] Department of Animal Science, Biotechnical Faculty, University of Ljubljana, Groblje 3, 1230 Domžale, Slovenia

[3] Sferogen d.o.o, Vipavska cesta 2c, 5270 Ajdovščina, Slovenia

[4] Sartorius BIA Separations, Mirce 21, 5270 Ajdovščina, Slovenia

[5] Valdoltra Orthopaedic Hospital, Jadranska cesta 31, 6280 Ankaran, Slovenia

[6] Faculty of Medicine, University of Ljubljana, Vrazov trg 2, 1000 Ljubljana, Slovenia

[#] Correspondence: Vida Štilec, vida.stilec@cobik.si

Supplementary Materials

1. **Figures: Supplementary Figure 1**
2. **ARRIVE Checklist Additional Details**
3. **ARRIVE Checklist**

1. Figures

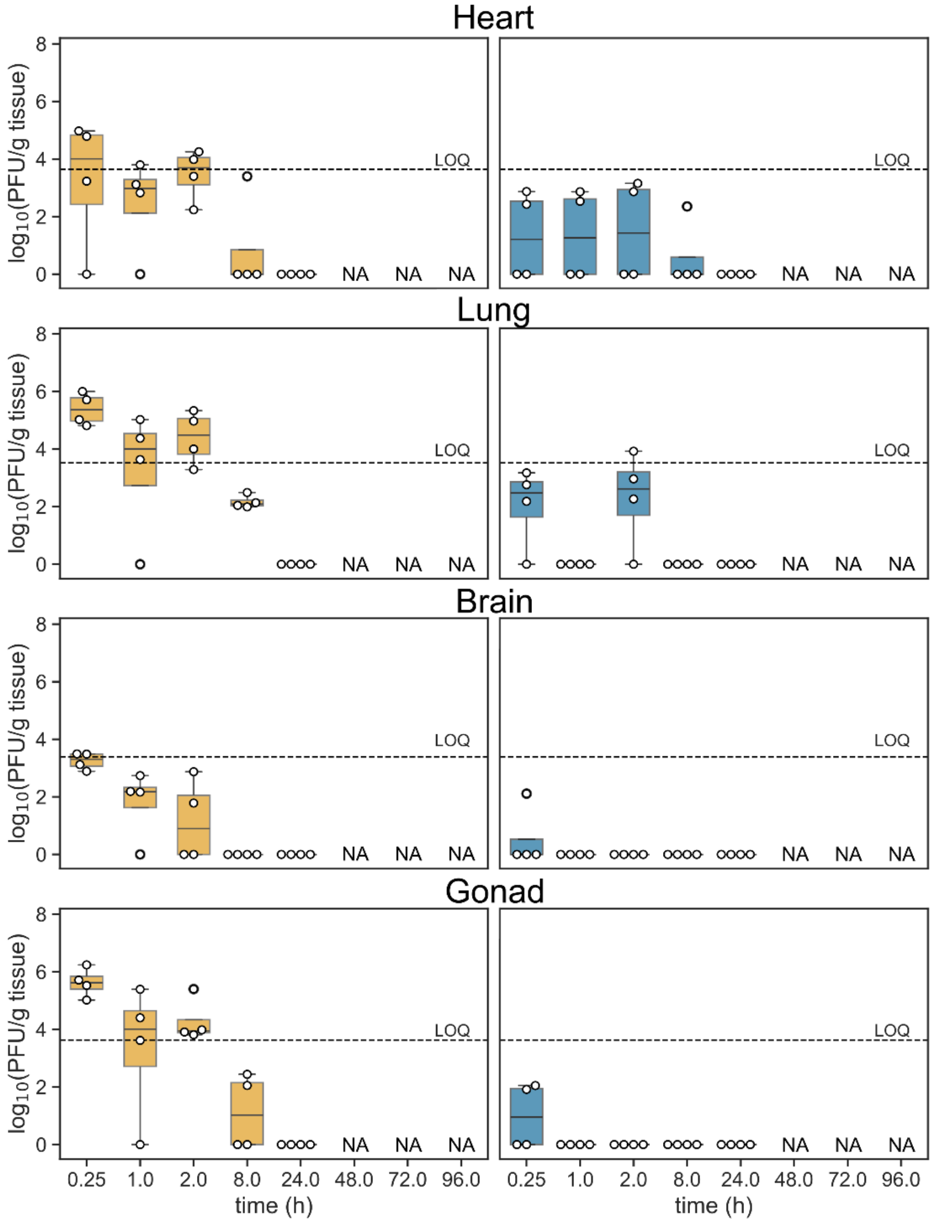

Supplementary Figure 1: Biodistribution of active phages in organs (heart, lung, brain and gonad) following a single IP (yellow) and IA (blue) administration in healthy mice. Active phage titer is expressed as PFU per gram of each organ; the LOQ for heart is 3.6 log_{10}PFU/g tissue, LOQ for lung 3.5 log_{10}PFU/g tissue, LOQ for brain 3.4 log_{10}PFU/g tissue and LOQ for gonad 3.6 log_{10}PFU/g tissue. Phage titer was determined by double agar overlay plaque assay, and values are plotted as boxplot with the box extending from the first quartile (Q1) to the third quartile (Q3) of the data, with a line at the median. The whiskers extend from the box to the farthest data point lying within 1.5x the inter-quartile range (IQR) from the box. Flier points are those past the end of the whiskers (N = 4). NA: not analyzed, PFU: plaque-forming unit.

2. ARRIVE Checklist Additional Details

Sample Size:

- 2b: Explain how the sample size was decided. Provide details of any a priori sample size calculation, if done.

Sample size was determined to be the minimal number yielding meaningful results and was based on our previous experience with immunological experiments on mice and on already published literature data.

Inclusion and Exclusion Criteria:

- 3a: Describe any criteria used for including and excluding animals (or experimental units) during the experiment, and data points during the analysis. Specify if these criteria were established a priori. If no criteria were set, state this explicitly.

A priori, all healthy male C57BL/6_OlaHsd mice, aged 8-10 weeks, were eligible for inclusion in our study. Exclusion criteria included animals with obvious signs of active infection (n = 0). No additional inclusion or exclusion criteria were set.

Randomization:

- 4b: Describe the strategy used to minimise potential confounders such as the order of treatments and measurements, or animal/cage location. If confounders were not controlled, state this explicitly.

To minimize confounders, all animals were ordered from the same vendor and housed in the same facility, and given the same environmental enrichment, food, and water. Materials from the same manufacturer were utilized for all procedures, and a single individual was responsible for phage application. The same batch of phage was used on all animals.

Blinding:

- 5: Describe who was aware of the group allocation at the different stages of the experiment (during the allocation, the conduct of the experiment, the outcome assessment, and the data analysis).

The study team (VŠ, SH) were aware of group allocations during the allocation and conduct of the experiment. Group allocations during outcome assessment and data analysis were known to part of the study team (VŠ and SH).

Experimental Animals:

- 8b: Provide further relevant information on the provenance of animals, health/immune status, genetic modification status, genotype, and any previous procedures.

All animals were obtained from Envigo (Envigo, Italy). We used a strain of healthy, non-immunocompromised C57BL/6_OlaHsd. No prior procedures were performed on these animals.

Background:

- 12b: Explain how the animal species and model used address the scientific objectives and, where appropriate, the relevance to human biology.

We used C57BL/6_OlaHsd mice due to institutional familiarity with this strain and availability of wide range of genetically modified strains developed on this genetic background which would be useful for potential further studies. We used mice in this study because of economic and practical reasons as we planned to test several different experimental conditions (two application routes, eight time points) resulting in a high number of animals needed (N = 108). Use of a mouse model enables us to compare phage biodistribution following two different application routes during 4-day period, which can help inform dosing and pharmacokinetic studies in larger animal models and human phage therapy applications.

Animal care and monitoring:

- 16c: Describe the humane endpoints established for the study, the signs that were monitored and the frequency of monitoring. If the study did not have humane endpoints, state this.

Animals were evaluated daily to determine their well-being by monitoring animal behavior and food and water consumption. Pain assessment was conducted using the Mouse Grimace Scale (MGS). If signs of changed well-being or pain appeared (apathy, hunched posture, rough or unthrifty hair coat, abnormal vocalization, skin necrosis/abscesses) the animals would be more frequently monitored and evaluated if the criteria for consideration of humane endpoints is reached. If the animal reached humane endpoints, it would be excluded from the experiments and euthanized. Humane endpoints included:

  - >20% weight loss
  - Dyspnea
  - Ulcer at the site of application
  - Permanent lack of movement
  - Bloody diarrhea

Data Access:

- 20: Provide a statement describing if and where study data are available.

Data from this study will be made available upon reasonable request by the corresponding author.

NOTE: Please save this file locally before filling in the table, DO NOT work on the file within your internet browser as changes will not be saved. Adobe Acrobat Reader (available free here) is recommended for completion.

The ARRIVE guidelines 2.0: author checklist

The ARRIVE Essential 10

These items are the basic minimum to include in a manuscript. Without this information, readers and reviewers cannot assess the reliability of the findings.

Item		Recommendation	Section/line number, or reason for not reporting
**Study design**	1	For each experiment, provide brief details of study design including:	
		a. The groups being compared, including control groups. If no control group has been used, the rationale should be stated.	Methods: PK study of COP-80B in vivo
		b. The experimental unit (e.g. a single animal, litter, or cage of animals).	Methods: Animals
**Sample size**	2	a. Specify the exact number of experimental units allocated to each group, and the total number in each experiment. Also indicate the total number of animals used.	Methods: PK study of COP-80B in vivo
		b. Explain how the sample size was decided. Provide details of any *a priori* sample size calculation, if done.	Supplementary Material
**Inclusion and exclusion criteria**	3	a. Describe any criteria used for including and excluding animals (or experimental units) during the experiment, and data points during the analysis. Specify if these criteria were established *a priori*. If no criteria were set, state this explicitly.	Supplementary Material
		b. For each experimental group, report any animals, experimental units or data points not included in the analysis and explain why. If there were no exclusions, state so.	No exclusions; Methods
		c. For each analysis, report the exact value of *n* in each experimental group.	See Results
**Randomisation**	4	a. State whether randomisation was used to allocate experimental units to control and treatment groups. If done, provide the method used to generate the randomisation sequence.	Methods: Animals
		b. Describe the strategy used to minimise potential confounders such as the order of treatments and measurements, or animal/cage location. If confounders were not controlled, state this explicitly.	Supplementary Material
**Blinding**	5	Describe who was aware of the group allocation at the different stages of the experiment (during the allocation, the conduct of the experiment, the outcome assessment, and the data analysis).	Supplementary Material
**Outcome measures**	6	a. Clearly define all outcome measures assessed (e.g. cell death, molecular markers, or behavioural changes).	Methods: Tissue harvest, processing and phage enumeration
		b. For hypothesis-testing studies, specify the primary outcome measure, i.e. the outcome measure that was used to determine the sample size.	N/A
**Statistical methods**	7	a. Provide details of the statistical methods used for each analysis, including software used.	Methods: Data interpretation and statistical analysis
		b. Describe any methods used to assess whether the data met the assumptions of the statistical approach, and what was done if the assumptions were not met.	Methods: Data interpretation and statistical analysis
**Experimental animals**	8	a. Provide species-appropriate details of the animals used, including species, strain and substrain, sex, age or developmental stage, and, if relevant, weight.	Methods: Animals
		b. Provide further relevant information on the provenance of animals, health/immune status, genetic modification status, genotype, and any previous procedures.	Supplementary Material
**Experimental procedures**	9	For each experimental group, including controls, describe the procedures in enough detail to allow others to replicate them, including:	
		a. What was done, how it was done and what was used.	a) Methods: Phage dosing and administration
		b. When and how often.	b) Methods: Phage dosing and administration
		c. Where (including detail of any acclimatisation periods).	c) Methods: Animals
		d. Why (provide rationale for procedures).	d) Methods: Phage dosing and administration
**Results**	10	For each experiment conducted, including independent replications, report:	
		a. Summary/descriptive statistics for each experimental group, with a measure of variability where applicable (e.g. mean and SD, or median and range).	Results
		b. If applicable, the effect size with a confidence interval.	N/A

The Recommended Set

These items complement the Essential 10 and add important context to the study. Reporting the items in both sets represents best practice.

Item		Recommendation	Section/line number, or reason for not reporting
**Abstract**	11	Provide an accurate summary of the research objectives, animal species, strain and sex, key methods, principal findings, and study conclusions.	Abstract
**Background**	12	a. Include sufficient scientific background to understand the rationale and context for the study, and explain the experimental approach.<br>b. Explain how the animal species and model used address the scientific objectives and, where appropriate, the relevance to human biology.	Introduction, Discussion<br>Supplementary Material
**Objectives**	13	Clearly describe the research question, research objectives and, where appropriate, specific hypotheses being tested.	Introduction, Discussion
**Ethical statement**	14	Provide the name of the ethical review committee or equivalent that has approved the use of animals in this study, and any relevant licence or protocol numbers (if applicable). If ethical approval was not sought or granted, provide a justification.	Methods
**Housing and husbandry**	15	Provide details of housing and husbandry conditions, including any environmental enrichment.	Methods: Animals
**Animal care and monitoring**	16	a. Describe any interventions or steps taken in the experimental protocols to reduce pain, suffering and distress.<br>b. Report any expected or unexpected adverse events.<br>c. Describe the humane endpoints established for the study, the signs that were monitored and the frequency of monitoring. If the study did not have humane endpoints, state this.	Methods: Animals, Phage dosing and administration<br>Results<br>Supplementary Material, Results
**Interpretation/ scientific implications**	17	a. Interpret the results, taking into account the study objectives and hypotheses, current theory and other relevant studies in the literature.<br>b. Comment on the study limitations including potential sources of bias, limitations of the animal model, and imprecision associated with the results.	Discussion<br>Discussion
**Generalisability/ translation**	18	Comment on whether, and how, the findings of this study are likely to generalise to other species or experimental conditions, including any relevance to human biology (where appropriate).	Discussion
**Protocol registration**	19	Provide a statement indicating whether a protocol (including the research question, key design features, and analysis plan) was prepared before the study, and if and where this protocol was registered.	Methods: Ethical approval
**Data access**	20	Provide a statement describing if and where study data are available.	Supplementary Material
**Declaration of interests**	21	a. Declare any potential conflicts of interest, including financial and non-financial. If none exist, this should be stated.<br>b. List all funding sources (including grant identifier) and the role of the funder(s) in the design, analysis and reporting of the study.	Declaration of Competing Interest<br>Funding
